# The interplay of semantic and syntactic processing across hemispheres

**DOI:** 10.1038/s41598-024-51793-2

**Published:** 2024-03-04

**Authors:** Sangyub Kim, Kichun Nam, Eun-Ha Lee

**Affiliations:** 1 Department of Psychology, Chonnam National University, 77, Yongbong-ro, Buk-gu, Gwangju, 61217 Republic of Korea; 2 Department of Psychology, Korea University, 145, Anam-ro, Seoungbuk-gu, Seoul, 02841 Republic of Korea; 3 Wisdom Science Center, Korea University, 145, Anam-ro, Seoungbuk-gu, Seoul, 02841 Republic of Korea

**Keywords:** Human behaviour, Language, Reading

## Abstract

The current study investigated the hemispheric dynamics underlying semantic and syntactic priming in lexical decision tasks. Utilizing primed-lateralized paradigms, we observed a distinct pattern of semantic priming contingent on the priming hemisphere. The right hemisphere (RH) exhibited robust semantic priming irrespective of syntactic congruency between prime and target, underscoring its proclivity for semantic processing. Conversely, the left hemisphere (LH) demonstrated slower response times for semantically congruent yet syntactically incongruent word pairs, highlighting its syntactic processing specialization. Additionally, nonword data revealed a hemispheric divergence in syntactic processing, with the LH showing significant intrahemispheric syntactic priming. These findings illuminate the intrinsic hemispheric specializations for semantic and syntactic processing, offering empirical support for serial processing models. The study advances our understanding of the complex interplay between semantic and syntactic factors in hemispheric interactions.

## Introduction

In the realm of human cognition, language serves as an indispensable conduit for interpersonal communication and knowledge acquisition. Specifically, reading emerges as a pivotal skill, not merely facilitating interaction but also enabling a deeper understanding of the world. Within this context, the comprehension of lexical semantics is of paramount importance. Information processing during reading is influenced by a myriad of factors, among which the processing of semantic and syntactic relationships between adjacent words stands as particularly crucial. Semantic processing entails an integrative approach that scrutinizes the congruence of semantic associations between successive words within a given context. Conversely, syntactic processing involves the examination of the grammatical relationships between adjacent words to ascertain their syntactic appropriateness. These two dimensions of information processing are intrinsically interrelated. The efficacy of semantic processing may be contingent upon syntactic consistency, as exemplified by the juxtaposition of adjectives and nouns in phrases like “beautiful beach” versus “beautifully beach.” On the other hand, the efficiency of syntactic processing can be influenced by semantic congruence, as illustrated by the pairing of adjectives and nouns in phrases such as “smart detective” compared to “turbid detective.” Thus, a comprehensive understanding of both semantic and syntactic relationships is imperative for optimizing the cognitive processes underlying reading comprehension.

Notwithstanding the extensive body of literature on linguistic processing, there exists a conspicuous gap in research concerning the hemispheric interactions that underlie semantic and syntactic processing. Language processing is inherently a dynamic neural activity, necessitating intricate interactions among various cerebral regions. Specifically, the bilateral macrostructures of the brain—namely, the left and right hemispheres—engage in collaborative efforts to facilitate a diverse array of linguistic functions. The corpus callosum, a critical neural structure, serves as the conduit for interhemispheric communication. This anatomical feature enables the dynamic exchange of information between the hemispheres^1,2^, thereby playing a pivotal role in the semantic and syntactic processing essential for reading comprehension^3,4^.

While a plethora of existing research underscores the left hemisphere’s (LH) predominance in linguistic processing^5–7^, the role of the right hemisphere (RH) remains a subject of nuanced debate. Although the RH has traditionally been ascribed a primary role in nonverbal information processing^8–10^, emerging evidence suggests its substantive involvement in linguistic functions as well^11–13^. For instance, studies involving commissurotomized patients—individuals whose interhemispheric connections have been surgically severed for the treatment of intractable epilepsy—indicate that the isolated RH retains capabilities for understanding both auditory and visual language across a range of lexical categories and semantic relationships^14^. Furthermore, neuroimaging studies employing positron emission tomography (PET) and functional magnetic resonance imaging (fMRI) have frequently reported bilateral cerebral activity during language comprehension tasks^15,16^. In addition, the RH has been implicated in specific aspects of language comprehension, including discourse analysis and inferential reasoning^17–20^. Thus, it is posited that the RH employs distinct strategies, particularly in the semantic processing of words. Conversely, the LH, especially regions such as the left inferior frontal gyrus—commonly known as Broca’s area—exhibits a more pronounced role in syntactic processing^21,22^. However, this specialization is not monolithic; task-specific requirements and stimulus manipulations can influence the activation patterns within the left inferior frontal gyrus, thereby introducing a degree of functional asymmetry between the hemispheres^23^. Given this dichotomy—wherein the LH predominantly governs syntactic processing and the RH largely oversees semantic processing—it is anticipated that complex interhemispheric interactions will manifest during the concurrent processing of semantic and syntactic elements in reading.

To examine the complexities of hemispheric interactions in reading comprehension, the current investigation focuses on the collaborative roles of the left and right hemispheres during the process of individual word recognition, employing the visual half-field presentation paradigm. In this experimental framework, stimuli are unilaterally presented in the visual field, thereby eliciting initial processing in the contralateral hemisphere. For example, stimuli presented in the left visual field (LVF) activate initial processing in the RH, while those in the right visual field (RVF) engage the LH. Leveraging the principles of visual half-field presentation, we employed successive stimulus presentations in the parafoveal vision at varying temporal intervals—50 ms^24^, 300 ms^25^, and 600 ms^3^—to induce dynamic interhemispheric interactions. Semantic manipulation of the prime and target words enabled the assessment of hemispheric semantic priming based on their unilateral visual field presentation. Specifically, when the prime and target were presented in opposing unilateral visual fields (e.g., prime in LVF/RH and target in RVF/LH), the study evaluated interhemispheric semantic priming predicated on semantic congruency. In contrast, when both prime and target were displayed in the same unilateral visual field (e.g., both in LVF/RH), intrahemispheric semantic priming was assessed. Similarly, the present study also gauged both intrahemispheric and interhemispheric syntactic priming, contingent upon the unilateral visual field of presentation and the syntactic congruence between the prime and target words. As a result, two distinct stimuli, bearing semantic and/or syntactic relationships, were sequentially presented in the parafoveal field. This methodology facilitated an evaluation of intra- and interhemispheric interactions, thereby offering invaluable insights into the hemispheric dynamics that underpin semantic and syntactic processing in reading.

Within the framework of successive stimulus presentation, two salient forms of priming—semantic and syntactic—emerge, each with distinct characteristics and underlying mechanisms. First, semantic priming, a phenomenon extensively investigated in prior research^26–29^, refers to the facilitative effect on semantic processing engendered by semantically congruent prime-target pairs. This facilitation manifests as expedited and more accurate lexical decisions when the prime and target exhibit semantic congruence, as opposed to when they are semantically incongruent. For example, a prime such as “scary” elicits quicker and more accurate lexical decisions for a congruent target like “bear” compared to an incongruent prime like “pencil.” Second, syntactic priming, alternatively termed grammatical priming, encompasses multiple dimensions of syntactic relationships between prime and target. These dimensions include gender and number agreement^30–32^, word category congruency^27,33^, and subject/auxiliary verb-verb correspondence^34^. Syntactic priming engenders a facilitative effect on syntactic processing when syntactically congruent prime-target pairs are presented. This results in accelerated and more accurate lexical decisions in comparison to syntactically incongruent pairs. For instance, prime such as “beautiful” yield faster and more accurate lexical decisions when the target is “beach,” as opposed to an incongruent syntactic prime like “beautifully.” Thus, both semantic and syntactic priming serve as invaluable tools for dissecting the intricate dynamics of hemispheric interactions in language processing, particularly in the realm of reading comprehension.

Moreover, the efficacy of semantic and syntactic priming may be modulated by the nature of intra- and interhemispheric interactions, owing to the asymmetric specialization of the two cerebral hemispheres. The conceptual framework of the current study was anchored in the notion that enhanced priming effects may be observed when stimulus presentation is strategically aligned with hemisphere-specific proficiencies in information processing. In the case of prime-target pairs that are semantically congruent but syntactically incongruent, this study posited that more robust semantic priming effects will be elicited when the prime is displayed in the LVF, thereby predominantly engaging the RH, as compared to alternative presentation modalities. Conversely, in instances where prime-target pairs are semantically incongruent yet syntactically congruent, elevated syntactic priming was anticipated when the prime is presented in the RVF, thereby activating the LH, with the target subsequently presented in the LVF to engage the RH. This framework sought to attribute the observed variances in priming effects—whether semantic or syntactic—to the specialized capabilities of the cerebral hemispheres; the RH is posited to excel in semantic processing, while the LH is understood to demonstrate a proficiency in syntactic processing.

### Theoretical models of semantic and syntactic processing

Extant literature has suggested two theoretical frameworks to explicate the mechanisms underlying visual word processing within semantically and syntactically congruent contexts. The first, known as the serial processing model, posits a hierarchical approach to linguistic comprehension. Initial processing is devoted to the establishment of syntactic representation, such as phrase structure, which predominantly relies on part-of-speech information. Subsequent stages involve lexical-semantic and morphological-syntactic processing, which facilitate the assignment of semantic roles, including the argument structures of verbs and gender/number agreement. Ultimately, semantic, pragmatic, and syntactic information are integrated into a coherent representation^27^. According to this serial model, syntactic processing exerts an influence on semantic processing, but not reciprocally. Empirical support for this model is derived from electrophysiological studies by Friederici^35^ and Friederici et al.^27^. In these studies, conditions devoid of semantic and syntactic congruence between the prime and target—such as semantic incongruities and violations of part-of-speech correspondence between prime and target—failed to elicit significant N400 effects. In accordance with the previous finding^36^, it is acknowledged that N400 effects are predominantly amplified in instances of semantic incongruity between prime and target. The observed lack of N400 effects in scenarios where there is an absence of both semantic and syntactic congruence between the prime and target suggests a preponderance of syntactic over semantic congruency. This led the researchers to posit that syntactic information serves as an early, independent reference point, uninfluenced by semantic processing. Additionally, in sentence anomaly detection tasks, the identification of semantic inconsistencies was found to be more time-consuming than the detection of syntactic anomalies, thereby reinforcing the validity of the serial processing model.

In contrast to the serial model, the parallel processing model posits that contextual information is initially processed through a bottom-up mechanism, activating lexical candidates irrespective of their semantic or syntactic attributes. Information processing, in this model, is contingent upon the availability of referable data, thereby rendering the sequence of semantic and syntactic processing flexible and, under certain conditions, simultaneous^29^. Empirical support for the parallel model is provided by van den Brink and Hagoort^29^, who observed earlier peaks than those associated with Left Anterior Negativity (LAN) in conditions where the target word was incongruent with the prime sentence, both semantically and syntactically (e.g., semantic discordance between the prime adjective and the target verb, and violation of part-of-speech correspondence). These findings were interpreted as evidence for the preferential utilization of available information, irrespective of its semantic or syntactic nature, thereby substantiating the parallel processing model. Given these contrasting theoretical frameworks, the present study endeavors to explore the interplay between syntactic and semantic processing within the context of hemispheric dynamics. Specifically, we aim to ascertain whether syntactic congruence modulates semantic processing, whether semantic congruence influences syntactic processing, or whether an interactive relationship exists between the two.

Therefore, this investigation employed a primed-lateralized lexical decision task to investigate the dynamics of semantic and syntactic priming in parafoveal lexical decision-making, utilizing congruency between prime and target. This approach facilitates an examination of the influence of hemispheric propagation sequences on these priming effects. The experimental design was structured to quantify semantic priming through the differential response latencies between semantically congruent and incongruent pairings. Similarly, syntactic priming was assessed by contrasting responses to syntactically congruent versus incongruent pairs. Subsequently, the current study explored the impact of hemispheric propagation sequences by manipulating the visual fields of prime and target within the parafoveal region, encompassing both left and right parafoveal vision.

In the context of our research, we suggested the following hypotheses: Firstly, predicated upon the postulation of distinct strategies employed by the RH in semantic processing^17–20^, we hypothesized that semantic priming will emanate from the LVF/RH and extend to targets situated within either the LVF or the RVF in parafoveal vision. Thus, this is projected to exert an impact on semantic processing across both the left and right cerebral hemispheres. Secondly, in light of the more pronounced role ascribed to the LH in syntactic processing^21,22^, we hypothesized that syntactic priming will originate from the RVF/LH to any target visual field, signifying that the LH will syntactically prime both the RVF/LH and LVF/RH. Additionally, we aimed to engage with existing theoretical frameworks concerning semantic and syntactic processing, particularly in the context of hemispheric interactions, to provide an interpretation of our findings. This approach not only tests the validity of our hypotheses but also contributes to the broader discourse on the complex interplay between semantic and syntactic processing across cerebral hemispheres.

## Method

### Participants

A total of 60 participants, comprising 18 males and 42 females, were recruited to partake in the current investigation. There were no instances of data exclusion as all participants adhered to the experimental protocol without encountering any complications. Handedness was assessed utilizing the Edinburgh Handedness Inventory^37^, revealing that all participants were right-handed (*M* = 8.2, *SD* = 1.63). The final data analysis included data from all participants with a mean age of 23.22 years (*SD* = 3.26). Normal or corrected-to-normal vision in both eyes was reported among all participants. Prior to participation, all individuals provided informed consent after comprehensive comprehension of the ethical guidelines approved by the Institutional Review Board of Korea University (KUIRB-2023-0250-01). All experimental protocols received approval from the Institutional Review Board of Korea University, the ethics committee of Korea University, South Korea, where the study was conducted. The study strictly conformed to the ethical tenets set forth in the 1964 Declaration of Helsinki. Participants with documented histories of neurological impairments resulting from brain injury or stroke were deemed ineligible and excluded from the experiment.

### Experimental task and procedure

**Figure 1:**
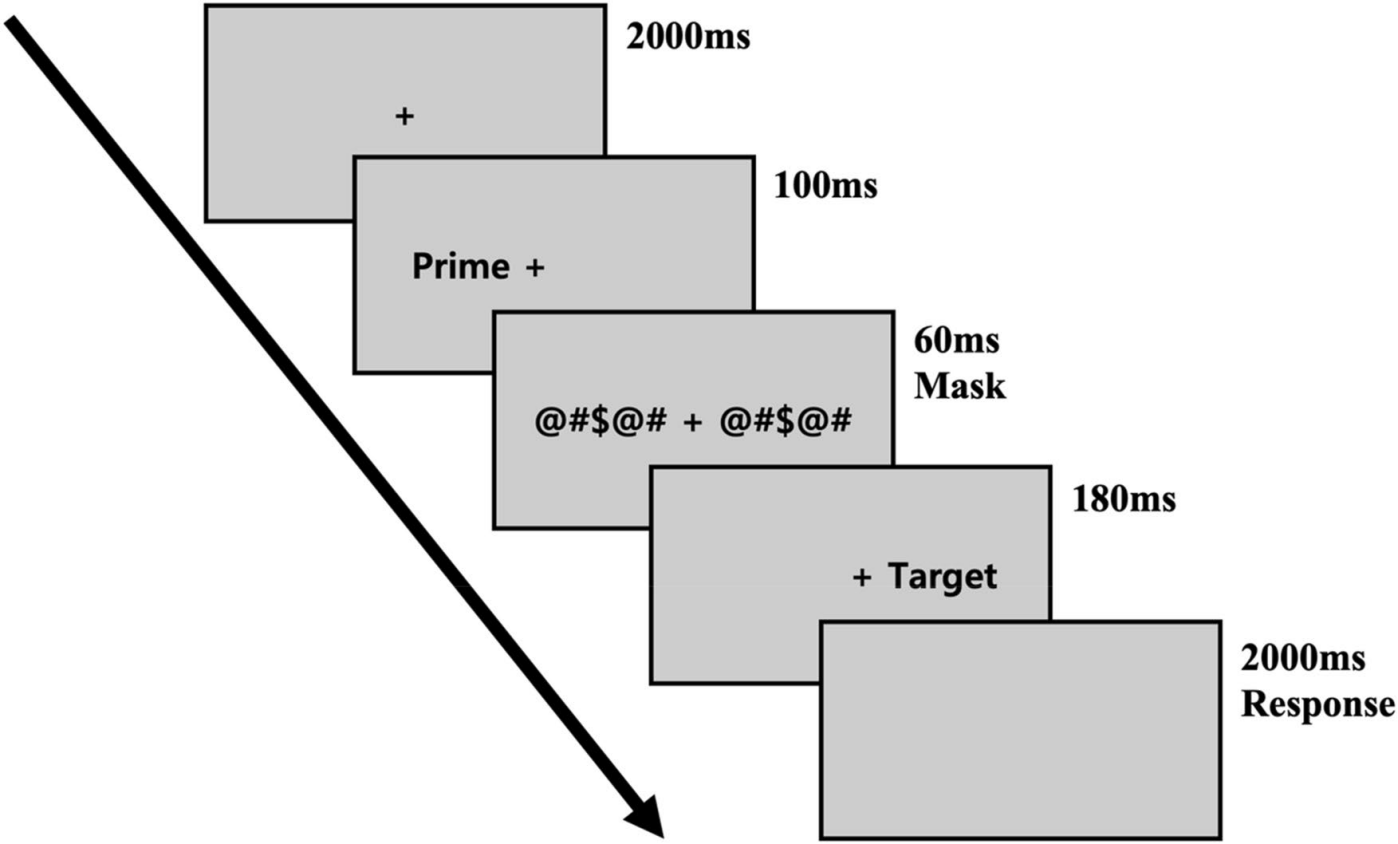
Schematic representation of the primed-lateralized lexical decision task paradigm in experiment.

The experimental paradigm employed in this study entailed a primed-lateralized lexical decision task, designed to investigate interhemispheric interactions for primed word recognition. During the task, participants were presented with visual stimuli (prime and target) in a sequential manner. Initially, a fixation point (‘+’) was displayed at the central vision for a duration of 2000 ms, serving as a visual anchor. This was followed by the presentation of a prime stimulus at the parafoveal vision, lasting for 100 ms. Subsequently, masks composed of nonsensical symbols (‘@#$@#’) were swiftly presented for 60 ms, effectively occluding the prime stimulus. Shortly after, a target stimulus appeared at the parafoveal vision for 180 ms. To capture participants’ responses, a black screen was then presented, providing a time window of 2000 ms within which participants indicated whether the target stimulus was a word or a nonword. Figure 1 visually depicted the sequential flow of the experimental procedure involved in the primed-lateralized lexical decision task implemented in this study.

### Experimental condition

To investigate the semantic and syntactic priming within the framework of hemispheric interactions, the current study employed a primed-lateralized lexical decision task featuring an array of designed experimental conditions. For words, four conditions were delineated: (1) Semantically and Syntactically Congruent Condition (referred to as the “*Correct*” or OO condition), exemplified by the prime-target pair “honest” and “conversation”; (2) Syntactically Violated Condition (referred to as the “*Syntactic Violation*” or OX condition), characterized by semantically congruent but syntactically incongruent adverb-noun combinations such as “deeply” and “sea”; (3) Semantically Violated Condition (referred to as the “*Semantic Violation*” or XO condition), involving syntactically congruent but semantically incongruent adjective-noun pairs like “turbid” and “detective”; and (4) Combined Violated Condition (referred to as the “*Combined Violation*” or XX condition), featuring both semantic and syntactic incongruities, as in “quite” and “balloon”.

In the context of nonwords, this study delineated two distinct experimental conditions. The first, termed the “Syntactically Violated Filler Condition” (or fX condition), is characterized by syntactically discordant adverb-nonword pairs, exemplified by combinations such as “honorably” and “frintion”. The second, known as the “Syntactically Unviolated Filler Condition” (or fO condition), consists of syntactically harmonious adjective-nonword pairs, like “clear” and “diacity”. The nonword conditions were specifically employed to isolate and assess the effects of syntactic processing, attributable to the inherent lack of semantic content in these nonwords. In addition, in the context of nonword target processing, we posited that participants would inherently predict a syntactic link between the prime and target, stemming from their awareness that the target functions as a noun, necessitating a lexical decision. This understanding likely led to an intuitive grasp of the potential syntactic pairings, specifically anticipating adjectives or adverbs as primes preceding a noun target. In linguistic structures, particularly in Korean and English, adjectives are typically positioned before nouns, establishing a syntactic congruence, whereas adverbs, when used as primes, result in syntactic incongruence with the target. Therefore, even with a brief prime exposure of 100 ms, participants are likely to intuitively recognize the syntactic incongruity in adverb-prime cases and congruity in adjective-prime instances. This understanding allows for the inference that, during nonword processing, participants subconsciously categorize adverb primes as syntactically incongruent and adjective primes as syntactically congruent. This paradigm enables the assessment of syntactic priming effects in nonword target pairings.

Furthermore, concerning the presented location of the prime and target, they were positioned within the left or right parafoveal field. This arrangement was implemented to facilitate the directed propagation of stimuli to each cerebral hemisphere, thereby establishing distinct prime visual field (PVF: LVF/RVF) and target visual field (TVF: LVF/RVF) conditions.

### Stimuli and apparatus

To generate semantically congruent and incongruent pairs of prime words and target words in both syntactically congruent and incongruent conditions, we conducted a norming study following the extraction of 150 semantically congruent and 150 incongruent pairs of prime words and 2-syllable target words from the Korean Sejong Corpus^38^. In the experiment of the norming study for stimuli extraction, a fixation point was presented for 1000 ms, followed by a pair of prime word and target word simultaneously displayed in the foveal vision for 500 ms. Participants were then given 3500 ms to provide their responses by selecting a score ranging from 1 (indicating the most semantically unnatural pair) to 7 (indicating the most semantically natural pair) using a mouse cursor on the screen. A total of 20 participants (7 males, 13 females) with an average age of 23.1 years (age range: 20–33 years) took part in the experiment. Each participant was asked to assess the semantic naturalness of 300 syntactically congruent prime-target pairs. This evaluation enabled us to categorize the stimuli into 144 semantically congruent pairs (with ratings above 5.1) and 144 semantically incongruent pairs (with ratings below 3.85), following the exclusion of responses with missing values and instances of overlap between prime and target words across the pairs. Subsequently, we constructed syntactically incongruent pairs from the latter half of each set by grammatical manipulation, yielding 72 pairs that were semantically congruent but syntactically incongruent, and 72 pairs that were both semantically and syntactically incongruent. Semantically congruent pairs were evaluated based on a likelihood ratio exceeding 3, which serves as an indicator of lexical association. On the other hand, semantically incongruent pairs were defined as those that are not cataloged in the Korean Sejong Corpus^38^.

In the final analysis of the norming study, in the syntactically congruent condition, we evaluated 72 pairs that scored above 5.8 points as semantically congruent pairs, and 72 pairs that scored below 3.85 points as semantically incongruent pairs. For the syntactically incongruent condition, we evaluated 72 pairs that scored above 5.1 points as semantically congruent pairs and 72 pairs that scored below 3 points as semantically incongruent pairs. To ensure control over variables, we regulated target word frequency in terms of semantic congruency (congruent/incongruent), syntactic congruency (congruent/incongruent), PVF, and TVF [*F*(1, 288) = 0.101, *p* = 0.751, $\eta^{2}$ < 0.001]. Additionally, we controlled the length of the prime (number of syllables) across semantic congruency, syntactic congruency, PVF, and TVF [*F*(1, 288) = 0.404, *p* = 0.526, $\eta^{2}$ = 0.001]. Moreover, we generated target nonwords by randomly combining syllables used in words from the Korean Sejong Corpus. These target nonwords were then combined with prime adjectives or adverbs, resulting in 144 pairs of prime adjective words and target nonwords, as well as 144 pairs of prime adverb words and target nonwords.

In our controlled experimental setting, visual stimuli were presented on an LG monitor (27MK400H) with RGB color display, boasting a resolution of 1920 × 1080 pixels and a refresh rate of 75 Hz. We maintained a consistent viewing distance of 65 cm for all participants by employing a chin rest, ensuring the nasion’s fixed separation from the display screen. Following well-established visual presentation principles^39,40^, we carefully calibrated the visual angles for stimulus presentation, falling within a horizontal range of 2°–5° and a vertical range of 1.5°. Our stimulus presentation sequence was randomized and executed using E-Prime 2.0 Professional software (Psychology Software Tools, Inc., Pittsburgh, PA, United States). The text was rendered in a 13-point dotumche font, presented in white against a black background. Participants were given explicit instructions to respond to the stimuli using a keyboard positioned in front of the monitor. They employed the right-hand index finger to press the “/(slash)” key for indicating “word” responses, while the left-hand index finger was used to press the “z” key to signify “nonwords.” To ensure fairness and impartiality, the assignment of response keys was scrupulously counterbalanced across all participants. The entire experimental session lasted approximately 35 min, with each participant successfully completing the task within this timeframe.

### Analysis

In our analysis, our primary focus was on examining priming effects as assessed through response times (RTs) rather than accuracy (ACC). Our objective was to examine the processing speed of interhemispheric communication in lexical decision tasks, emphasizing the promptness of lexical decision-making over the precision of those decisions, in line with previous research (e.g., Kim et al.^3^). To initiate our investigation, we employed a one-sample t-test to ascertain the significance of the priming effects. This step was crucial to establish the presence of semantic and syntactic priming effects across the two hemispheres. Without confirming the significance of the priming effects, further interpretation would be premature. Subsequently, we conducted a comprehensive examination using a two-way repeated-measures analysis of variance (rm-ANOVA) with the factors of PVF (LVF/RVF) and TVF (LVF/RVF). This analysis aimed to investigate whether the observed semantic and syntactic priming effects were influenced by the hemispheric specialization, with the left hemisphere primarily responsible for syntactic processing and the right hemisphere for semantic processing. We employed two distinct measurements, namely XO-OO and OX-XX measurements for semantic priming effects, and OX-OO and XO-XX measurements for syntactic priming effects. Furthermore, we extended our analysis to the realm of nonword visual processing. This allowed us to examine syntactic priming effects in the absence of semantic processing while considering the hemispheric specialization. We utilized rm-ANOVA with a single measurement (fO-fX) for this specific investigation.

## Results

We gathered data on RTs and ACC in the primed-lateralized lexical decision task. No data were excluded from the final analysis, as all participants’ RTs for each experimental condition fell within three standard deviations. However, items with a mean ACC rate below 50% were excluded, affecting less than 3.5% of the overall data set. Table 1 present the behavioral responses, detailing both RTs and ACC across the experimental conditions.

**Table 1:** Behavioral response summary within each Experiment 1 condition, depicted in terms of RTs and ACC.

Lexicality	Congruency	Prime	Target	RTs	ACC
Words	OO	LVF	LVF	489.66 (120.29)	0.80 (0.16)
			RVF	512.64 (124.60)	0.70 (0.20)
		RVF	LVF	554.75 (137.56)	0.66 (0.19)
			RVF	461.04 (115.15)	0.85 (0.14)
	OX	LVF	LVF	485.17 (118.17)	0.78 (0.18)
			RVF	516.70 (130.38)	0.69 (0.18)
		RVF	LVF	548.81 (147.61)	0.69 (0.16)
			RVF	465.14 (117.09)	0.82 (0.12)
	XO	LVF	LVF	484.82 (122.45)	0.80 (0.17)
			RVF	548.07 (146.35)	0.70 (0.17)
		RVF	LVF	546.65 (140.76)	0.59 (0.17)
			RVF	475.57 (107.95)	0.81 (0.15)
	XX	LVF	LVF	504.42 (115.93)	0.76 (0.15)
			RVF	534.39 (140.08)	0.63 (0.16)
		RVF	LVF	532.69 (136.16)	0.68 (0.17)
			RVF	481.49 (112.68)	0.82 (0.14)
Nonwords	fO	LVF	LVF	564.09 (133.09)	0.78 (0.16)
			RVF	584.53 (150.66)	0.73 (0.14)
		RVF	LVF	589.77 (149.69)	0.73 (0.16)
			RVF	562.36 (141.32)	0.75 (0.14)
	fX	LVF	LVF	563.52 (135.35)	0.78 (0.14)
			RVF	582.44 (156.46)	0.72 (0.16)
		RVF	LVF	591.15 (149.83)	0.74 (0.14)
			RVF	550.51 (132.17)	0.81 (0.10)

Bracketed values signify corresponding standard deviations. Congruency categories for words include OO (semantically and syntactically congruent), OX (semantically congruent and syntactically incongruent), XO (semantically incongruent and syntactically congruent), and XX (semantically and syntactically incongruent). For nonwords, congruency categories encompass fO (syntactically congruent) and fX (syntactically incongruent).

Semantic and syntactic priming effects for words, as well as syntactic priming effects for nonwords, were described in Table 2. Additionally, the results of one-sample t-tests for each priming effect are presented in Table 2 to evaluate the statistical significance of the observed effects. The one-sample t-test revealed significant semantic priming in words, as assessed by the OX-XX measurement, involved the subtraction of RTs in the XX condition (semantically and syntactically incongruent) from those in the OX condition (semantically congruent yet syntactically incongruent), when the prime was displayed in the LVF/RH and the target in the RVF/LH [*t*(59) = − 2.07, *p* = 0.04], and vice versa [*t*(59) = 2.17, *p* = 0.03]. Furthermore, significant syntactic priming in nonwords was observed, as assessed by the fO-fX measurement, when both the prime and target were presented in the RVF/LH [*t*(59) = 2.55, *p* = 0.01].

**Table 2:** Comprehensive summary of semantic and syntactic priming effects within experimental conditions for words and syntactic priming effect for nonwords.

Lexicality	Type	Measurement	Prime	Target	Priming effect	T statistics	p-value
Words	Semantic priming	XO-OO	LVF	LVF	− 0.22 (77.89)	*t*(59) = − 0.02	0.98
				RVF	20.58 (98.11)	*t*(59) = 1.62	0.11
			RVF	LVF	− 18.67 (101.00)	*t*(59) = − 1.43	0.16
				RVF	11.18 (78.96)	*t*(59) = 1.10	0.28
		OX-XX	LVF	LVF	− 16.81 (86.58)	*t*(59) = − 1.50	0.14
				RVF	− 21.36 (80.00)	*t*(59) = − 2.07	0.04*
			RVF	LVF	22.78 (81.37)	*t*(59) = 2.17	0.03*
				RVF	− 9.08 (75.35)	*t*(59) = − 0.93	0.35
	Syntactic priming	OX-OO	LVF	LVF	− 4.94 (66.24)	*t*(59) = − 0.58	0.57
				RVF	− 7.74 (73.01)	*t*(59) = − 0.82	0.42
			RVF	LVF	12.67 (91.49)	*t*(59) = 1.07	0.29
				RVF	− 2.06 (77.41)	*t*(59) = − 0.21	0.84
		XO-XX	LVF	LVF	− 12.09 (70.96)	*t*(59) = − 1.32	0.19
				RVF	6.95 (92.06)	*t*(59) = 0.58	0.56
			RVF	LVF	− 8.56 (88.47)	*t*(59) = − 0.75	0.46
				RVF	4.16 (76.38)	*t*(59) = 0.42	0.67
Nonwords	Syntactic priming	fO-fX	LVF	LVF	− 1.23 (59.85)	*t*(59) = − 0.16	0.87
				RVF	2.45 (50.60)	*t*(59) = 0.37	0.71
			RVF	LVF	− 9.60 (86.44)	*t*(59) = − 0.86	0.39
				RVF	18.32 (55.54)	*t*(59) = 2.55	0.01*

Semantic priming effect calculated by subtracting RTs of XO from RTs of OO and subtracting RTs of OX from RTs of XX conditions in words. Syntactic priming effect for words determined by subtracting RTs of OX from RTs of OO and subtracting RTs of XO from RTs of XX conditions. Syntactic priming effect for nonwords measured by subtracting RTs of fO from RTs of fX conditions. Values in brackets indicate corresponding standard deviations. (*p < 0.05).

### Evaluation of semantic priming effect in words

**Figure 2:**
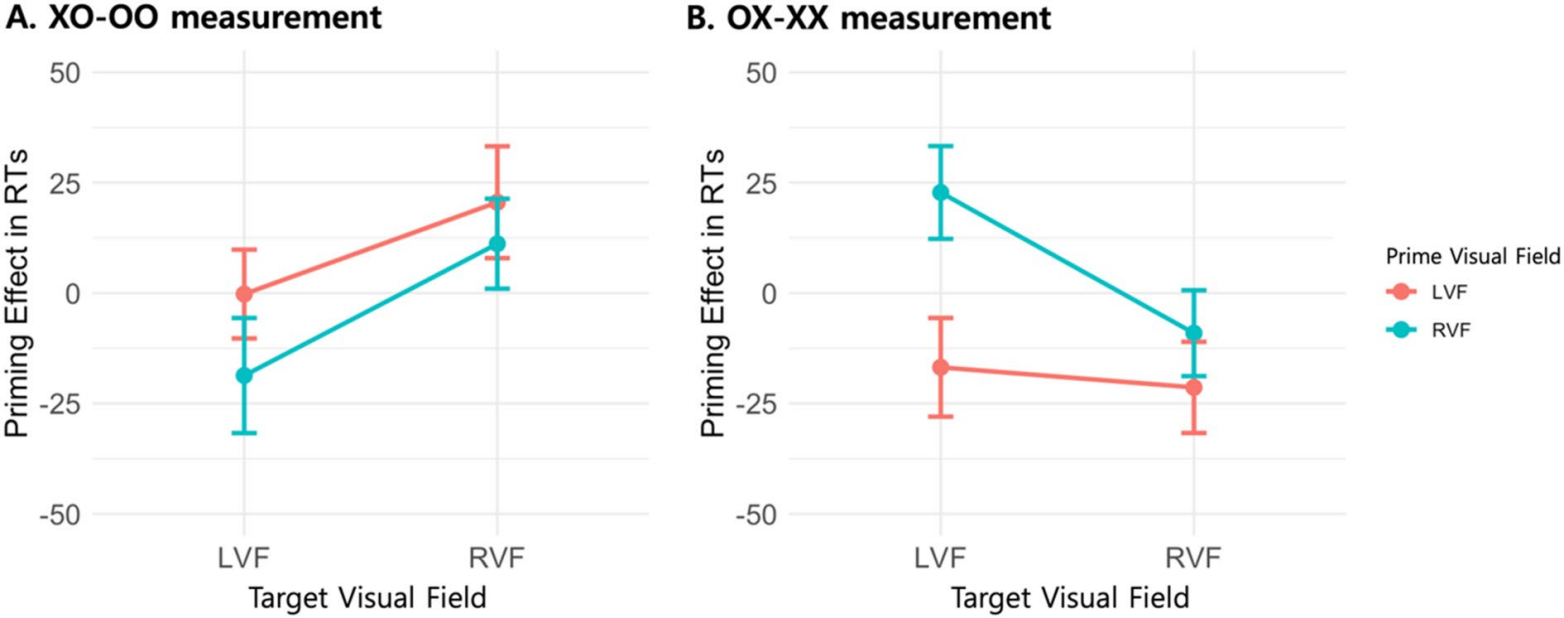
Illustration of semantic priming in words evaluated by XO-OO measurement (Panel **A**) and OX-XX measurement (Panel **B**). The line in the bar indicates standard error.

The semantic priming effect for words was assessed using two distinct measurements and described in Fig. 2. The initial measurement, denoted as XO-OO, involved the subtraction of RTs in the OO condition (semantically and syntactically congruent) from those in the XO condition (semantically incongruent yet syntactically congruent). This measurement allowed us to isolate the influence of semantic congruency between the prime and target while maintaining syntactic congruency. To evaluate the semantic priming effect ascertained by this first measurement across different visual fields, a two-way rm-ANOVA was conducted for each participant. The factors considered were the PVF (LVF/RVF) and the TVF (LVF/RVF). The analysis yielded no significant main effect for PVF [*F*(1, 59) = 0.427, *p* = 0.516, $\eta^{2}$ = 0.003] or TVF [*F*(1, 59) = 3.929, *p* = 0.052, $\eta^{2}$ = 0.018]. Moreover, the two-way interaction between PVF and TVF was also found to be non-significant [*F*(1, 59) = 0.423, *p* = 0.518, $\eta^{2}$ = 0.001].

In addition, we utilized a secondary evaluative parameter, labeled as OX-XX, to differentiate the RTs in the XX condition—marked by both semantic and syntactic incongruence—from those in the OX condition, characterized by semantic congruence but syntactic discordance. This approach allowed for the disentanglement of semantic priming effects in scenarios where syntactic incongruence existed between the prime and target. A two-way rm-ANOVA was executed for each participant, incorporating the variables of PVF (LVF/RVF) and TVF (LVF/RVF). The analysis revealed a significant main effect for PVF [*F*(1, 59) = 8.143, *p* = 0.006, $\eta^{2}$ = 0.041], while the main effect for TVF was not statistically significant [*F*(1, 59) = 1.358, *p* = 0.249, $\eta^{2}$ = 0.004]. Moreover, no significant two-way interaction between PVF and TVF was observed [*F*(1, 59) = 3.446, *p* = 0.068, $\eta^{2}$ = 0.015]. The pronounced main effect for PVF indicates accelerated RTs for semantically congruent yet syntactically incongruent prime-target word pairs when the prime was presented in the LVF/RH, in contrast to decelerated RTs under similar conditions when the prime was displayed in the RVF/LH.

### Evaluation of syntactic priming effect in words

**Figure 3:**
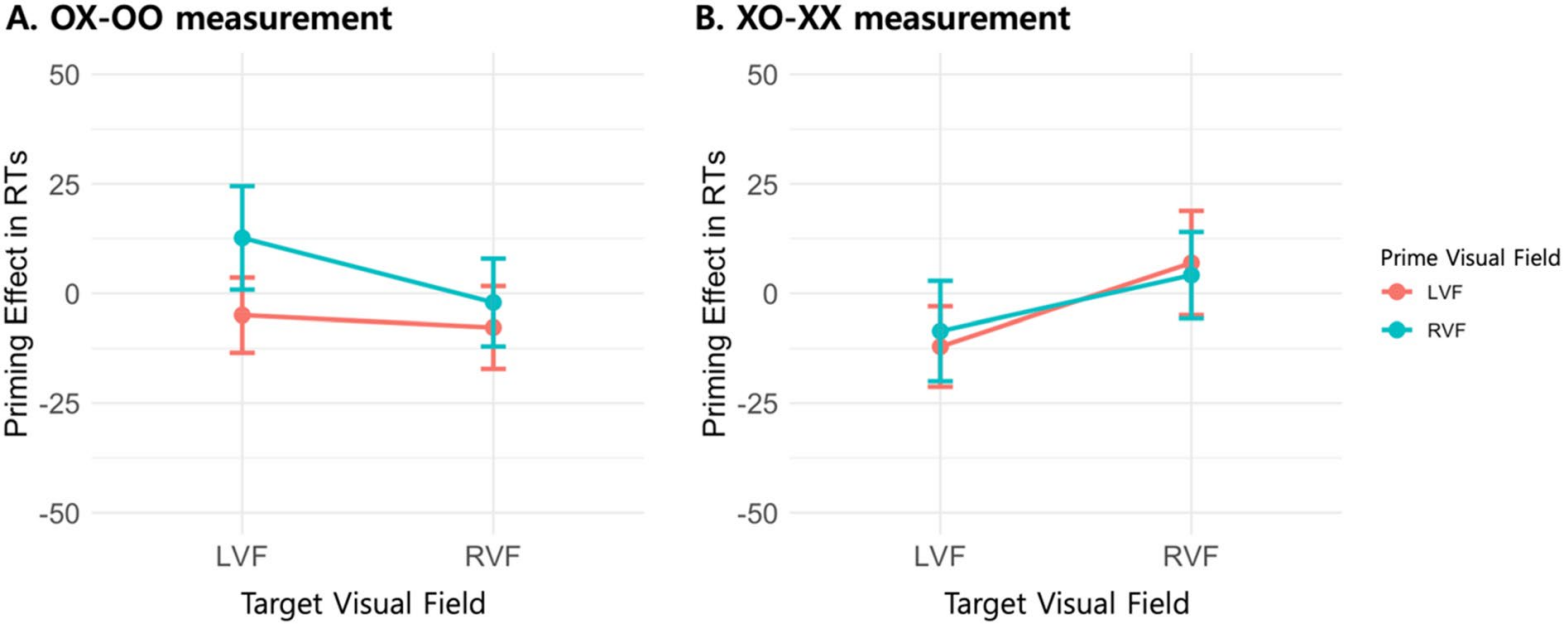
Visual representation of syntactic priming effects in words evaluated by OX-OO measurement (Panel A) and XO-XX measurement (Panel B). The line in the bar indicates standard error.

In the assessment of syntactic priming effects for words (see Fig. 3), two evaluative parameters were employed. The initial parameter, designated as OX-OO, involved subtracting the RTs in the OO condition—characterized by both semantic and syntactic congruence—from those in the OX condition, which featured semantic congruence but syntactic incongruence. This approach facilitated the isolation of syntactic priming effects in the context of syntactic incongruence between the prime and target, while maintaining semantic congruence. To quantify the syntactic priming effect as determined by this initial parameter, a two-way rm-ANOVA was conducted for each participant. This analysis incorporated factors of PVF (LVF/RVF) and TVF (LVF/RVF). The results indicated no significant main effect for either PVF [*F*(1, 59) = 2.689, *p* = 0.106, $\eta^{2}$ = 0.012] or TVF [*F*(1, 59) = 0.746, *p* = 0.391, $\eta^{2}$ = 0.003]. Additionally, the two-way interaction between PVF and TVF was found to be non-significant [*F*(1, 59) = 0.536, *p* = 0.467, $\eta^{2}$ = 0.002].

In evaluating syntactic priming effects, a secondary parameter, termed XO-XX, was employed. This involved subtracting the RTs in the XX condition—characterized by both semantic and syntactic incongruence—from those in the XO condition, which featured semantic incongruence but syntactic congruence. This approach allowed for the isolation of syntactic priming effects under conditions where both the prime and target were syntactically incongruent but semantically congruent. To quantify these effects, a two-way rm-ANOVA was conducted for each participant, incorporating factors of PVF (LVF/RVF) and TVF (LVF/RVF). The analysis yielded no significant main effect for PVF [*F*(1, 59) = 0.636, *p* = 0.428, $\eta^{2}$ = 0.003] or TVF [*F*(1, 59) = 3.271, *p* = 0.076, $\eta^{2}$ = 0.014]. Furthermore, the two-way interaction between PVF and TVF was found to be non-significant [*F*(1, 59) = 0.878, *p* = 0.353, $\eta^{2}$ = 0.003].

### Evaluation of syntactic priming effect in nonwords

**Figure 4:**
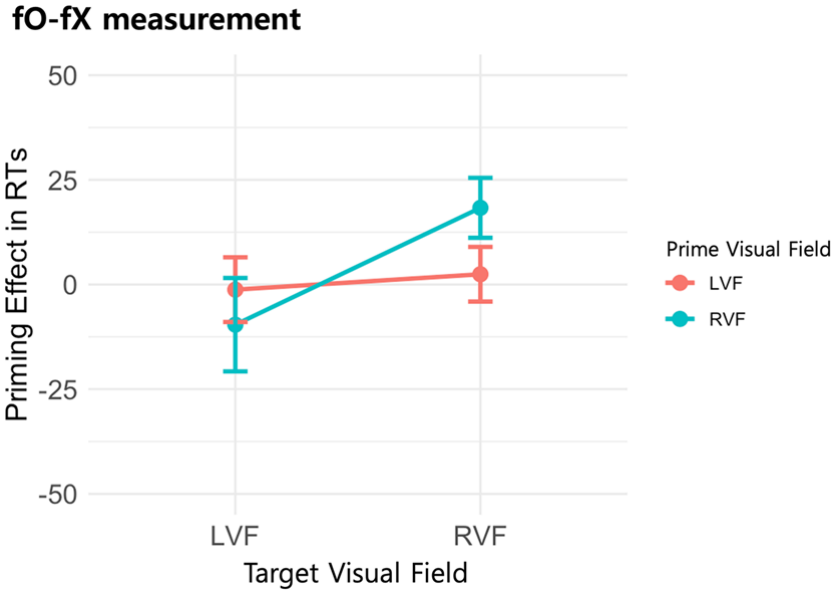
Demonstration of syntactic priming in nonwords evaluated by fO-fX measurement with standard error indicated in the bar.

To investigate the syntactic priming effect in nonwords (see Fig. 4), we employed a designated parameter, fO-fX, allowing us to isolate the influence of syntactic incongruency between the prime and target. A two-way rm-ANOVA was conducted for each participant, incorporating factors of PVF (LVF/RVF) and TVF (LVF/RVF). The analysis yielded a non-significant main effect for PVF [*F*(1, 59) = 0.169, *p* = 0.683, $\eta^{2}$ = 0.001], contrasted by a significant main effect for TVF [*F*(1, 59) = 4.699, *p* = 0.034, $\eta^{2}$ = 0.015]. No significant two-way interaction between PVF and TVF was observed [*F*(1, 59) = 1.809, *p* = 0.184, $\eta^{2}$ = 0.009]. The significant main effect for TVF suggests that reaction times were faster for syntactically congruent prime-target nonword pairs compared to their incongruent counterparts when the target was presented in the LVF/RH, while the opposite was true when the target was presented in the RVF/LH.

## Discussion

The current investigation sought to scrutinize semantic and syntactic priming within the framework of hemispheric dynamics, employing the primed-lateralized lexical decision task as the methodological instrument. To assess semantic priming, we utilized two distinct measurements—namely, XO-OO and OX-XX—owing to the potential modulatory influence of syntactic congruency between the prime and target on semantic priming outcomes. Concurrently, syntactic priming was evaluated through two specific measurements—OX-OO and XO-XX—to account for the possible impact of semantic congruency between the prime and target on syntactic priming phenomena. Our findings yielded multiple insights. First, upon evaluating semantic priming, we observed a significant main effect for PVF in the OX-XX measurement. Specifically, RTs were slower for OX pairs compared to XX pairs when the prime was presented in the RVF/LH. Conversely, faster RTs were recorded for OX pairs relative to XX pairs when the prime was displayed in the LVF/RH. Second, our data revealed no significant main or interaction effects between PVF and RVF across both syntactic priming measures (OX-OO and XO-XX).

### Hemispheric contributions to semantic priming in lexical decision tasks for words

In our analysis of semantic priming for lexical items, the significant main effect of PVF was observed exclusively in the OX-XX metric. This manifested as slower RTs for OX compared to XX when the prime was presented in the RVF/LH, in contrast to faster RTs for OX compared to XX when the prime was in the LVF/RH. Notably, this main effect of PVF was predominantly attributable to interhemispheric semantic priming effects, as opposed to intrahemispheric effects, as revealed by a one-sample t-test (Table 2). This suggests that the effect originates from responses where the target was presented in the LVF/RH following a RVF/LH prime, and vice versa. These findings imply a hemisphere-specific strategy for semantic processing. Specifically, semantic congruence does not facilitate semantic priming in the presence of syntactic incongruence for the LH, whereas it significantly enhances semantic priming irrespective of syntactic incongruence when the RH is primed. This suggests that the LH prioritizes syntactic correspondence prior to semantic priming, while the RH is less concerned with syntactic congruence, activating target responses as long as a semantic relationship with the prime exists.

Our findings intimated that inherent hemispheric disparities in semantic processing may engender divergent strategies in semantic priming. Prior research has posited the LH predilection for lexical association processing, contrasted with the RH bias for categorical information processing, thereby delineating a strategic dichotomy in semantic processing across the bilateral hemispheres. For instance, Grose-Fifer and Deacon (2004)^41^ examined facilitation for categorically-related word pairs with varying degrees of feature overlap in functional and physical attributes (e.g., “Boat–Tram” for Low feature overlap; “Tricycle–Tractor” for High feature overlap) and concluded that the RH’s proficiency in processing categorical pairs was attributable to shared features. Specifically, N400 facilitation was observed solely for pairs with high feature overlap and exclusively during LVF/RH presentation. Complementary studies have similarly failed to identify N400 priming for categorically related but lexically unassociated word pairs in the RVF/LH^42^. Moreover, patients with RH damage have been shown to lack a categorical priming effect on the N400^43^. These observations suggest that the RH plays a pivotal role in associating words that share features but are not lexically co-occurrent, potentially expediting responses for semantically congruent yet syntactically incongruent prime-target pairs in our study. Given the RH's capacity to strategically forge semantic links between the prime and target, irrespective of syntactic congruency, a significant differential in semantic priming effects was observed depending on whether the prime was presented in the LVF/RH or RVF/LH in the current investigation.

Conversely, the LH appears to be hindered in generating semantic priming by the presence of syntactic incongruency between the prime and target. This is evidenced by the observed the significant semantic priming when the prime was presented in the RVF/LH in the current study, manifesting as slower RTs for semantically congruent yet syntactically incongruent conditions compared to conditions that were both semantically and syntactically incongruent. We infer from this that the LH may facilitate semantic priming to the target exclusively when syntactic congruency exists between the prime and target, a predilection likely rooted in the LH's inherent specialization for syntactic processing. The pivotal role of syntactic processing in the LH is corroborated by extant literature. Empirical evidence suggests that the left inferior frontal gyrus, commonly known as Broca’s area, plays a significant role in syntactic processes, as demonstrated by both neuropsychological and neuroimaging studies^44,45^. Patients with lesions in the pars opercularis or pars triangularis of the left inferior frontal gyrus exhibit increased comprehension errors on reversible sentences devoid of semantic or pragmatic cues for thematic role assignments (e.g., “The girl that the boy is chasing is tall”), compared to sentences with such cues (e.g., “The man that the horse is riding is fat.”)^46^. These patients also manifest deficits in processing function words crucial for demarcating syntactic phrase boundaries and assigning thematic roles to lexical items^47^. While these observations underscore the centrality of Broca’s area in the LH for syntactic comprehension deficits, it is worth noting that other brain regions, including temporal and parietal areas, have also been implicated in syntactic processing^48–50^.

In light of the findings from this study and existing literature, a notable disparity in the strategies employed for semantic priming becomes apparent when comparing the two cerebral hemispheres. This investigation reveals that the RH is capable of eliciting semantic priming independently of the syntactic congruency or incongruency between the prime and target. Conversely, the LH appears reliant on syntactic congruence as a foundational element for the semantic priming. Once syntactic congruency is confirmed, semantic priming in the LH becomes evident. Prior research supports the accessibility of semantic information in the RVF/LH^51–53^. Additional studies have underscored the involvement of both hemispheres in semantic processing^4,54^, thus affirming the LH's ability in semantic information processing. However, our results indicate a unique dichotomy: while both hemispheres are capable of semantic processing, the LH's function is modulated or limited by syntactic variables, in contrast to the RH. This distinction offers novel insights into the differential mechanisms of semantic processing across hemispheres. In addition, this aligns with the assumptions inherent in serial models of semantic and syntactic processing, underscoring the importance of syntactic congruency as an initial step before semantic priming occurs. These insights suggest that differential hemispheric specialization gives rise to differential patterns of semantic priming.

### Hemispheric dynamics in syntactic priming for lexical decisions across words and nonwords

In our analysis of syntactic priming for lexical items, the rm-ANOVA revealed no significant main effects or interaction effects between PVF and TVF across both measurement conditions (OX-OO and XO-XX). Contrary to expectations, if the LH were predominantly responsible for syntactic processing, a differential pattern of hemispheric interaction in syntactic priming should have emerged based on the visual fields in which the prime and target were presented. However, the absence of such a pattern, despite well-documented evidence for focal syntactic processing in the LH^44,45,55^, suggests that syntactic processing at the lexical level may necessitate concurrent activation of both hemispheres. This is in line with findings by Lee et al.^56^, who employed foveal presentation for both the prime and target, thereby facilitating concurrent hemispheric activation. Despite the prime's brief 42 ms duration—insufficient for conscious perception—they observed significant syntactic priming. This suggests that foveal presentation may effectively engage both hemispheres in syntactic processing, thereby implicating a collaborative hemispheric strategy in the facilitation of syntactic priming.

In the context of nonwords, our analysis revealed a significant main effect for TVF, indicating slower RTs for syntactically congruent nonword pairs compared to their incongruent counterparts when the target was presented in the LVF/RH. Further analysis, as evidenced by a one-sample t-test (Table 2), pinpointed the source of this effect to significant syntactic priming when both the prime and target nonwords were presented in the RVF/LH. Given our earlier discussion on the LH’s proclivity for syntactic processing, this observed syntactic priming in nonwords can be attributed to the LH’s inherent strategy for primary syntactic processing. It appears that the LH, influenced by syntactic congruency between the prime and target, may erroneously categorize nonword primes and nonword targets as linguistically valid constructs, thereby resulting in slower RTs for syntactically congruent pairs compared to incongruent ones. This leads to a significant syntactic priming effect for nonwords when both the prime and target are presented in the RVF/LH.

For lexical decisions involving words, the complexity may extend beyond the focal syntactic processing domain in the LH, necessitating intricate intra- and interhemispheric interactions. Lesion studies have shown that patients with lesions outside of Broca's area in the LH exhibit similar syntactic processing deficits^48,49^, suggesting a more distributed neural network for syntactic processing in words. Furthermore, Positron Emission Tomography (PET) studies have indicated hypometabolism in temporal and parietal regions, rather than exclusively in Broca's area, in aphasic patients with comprehension deficits^50^. Task-based PET studies have also failed to localize syntactic processing to a single region within the inferior frontal area^57,58^, implicating a broader network involving both hemispheres in syntactic processing for words. This suggests that the RH may also play a role in syntactic processing, complementing the primary syntactic functions localized in the LH’s inferior frontal area. Conversely, for lexical decisions involving nonwords, focal syntactic processing within the LH may suffice for syntactic representation, obviating the need for significant contributions from the RH. This could result in slower RTs for syntactically congruent prime-target nonword pairs compared to incongruent pairs. The slower times may be attributed to intrahemispheric interactions within the LH, where confusion arises due to syntactic congruency between the prime and target nonwords.

### Hemispheric processing in theoretical frameworks of semantic and syntactic priming

The current study's findings lend partial support to serial models of semantic and syntactic processing. According to these models, semantic role assignment is predicated upon the establishment of syntactic structure. Our observations corroborated this notion, as significant semantic priming was only evident when the syntactic relationship between the prime and target was incongruent. This suggested a modulatory effect of syntactic relationships on semantic priming, aligning with the serial models' assumption of a sequential processing hierarchy, wherein syntactic structure representation precedes semantic representation. However, our study also revealed a noteworthy exception: significant semantic priming was observed even in conditions of syntactic incongruency (as measured by the OX-XX paradigm). This suggested that semantic integration can occur despite the absence of a stable syntactic relationship between the prime and target. Such a finding implied that semantic processing may not be entirely contingent upon prior syntactic structuring, thereby offering only partial support for the serial models.

However, the results of the current study indicated that the dichotomy in semantic and syntactic processing can be ascribed to the specialized capabilities of each cerebral hemisphere. In terms of semantic priming, we observed a facilitative effect in semantically congruent pairs—even when they were syntactically incongruent—when the prime was displayed in the LVF/RH and the target was presented in the RVF/LH. Conversely, an inhibitory semantic priming effect was observed in semantically incongruent pairs—even if syntactically incongruent—when the prime was oriented in the RVF/LH and the target appeared in the LVF/RH. These contrasting priming effects—facilitative in the context of RH prime presentation and inhibitory within the LH—underscored the specialized roles of each hemisphere in semantic and syntactic processing. These roles may, in turn, modulate the extent of semantic processing, emerging from a coordinated interhemispheric interaction that leverages these distinct specializations. Consequently, these findings added a layer of complexity to our understanding of semantic and syntactic processing within an overarching framework of interhemispheric cooperation. This necessitated a reevaluation of existing theoretical models pertaining to semantic and syntactic processes, particularly in conditions where syntactic structures are either ambiguous or clearly delineated. The implications of these results beckoned further investigations into the interplay between semantic and syntactic processing across varying levels of syntactic clarity.

### Possible emergence of hemispheric difference due to reading direction

In the context of normal reading, ocular fixations typically progress from left to right, thereby directing RVF stimuli initially to the LH. This lateralization necessitates a form of analytic processing in the LH, particularly for syntactic operations, as the gaze continues to move rightward to facilitate ongoing reading. Such a mechanism may provide the foundation for the LH's adeptness in managing syntactic operations. Specifically, during left-to-right reading, initial perception of right parafoveal words is projected to the LH, triggering a sequence of primary syntactic processing events upon the word's inaugural entry into the visual field via the right parafoveal region. Conversely, as the reading gaze continues to traverse leftward, words eventually transition into the LVF, thereby entering the RH via the human visual pathway. This dynamic may necessitate a form of semantic integration in the RH for words that have sequentially traversed the visual field. The RH, therefore, may prioritize semantic coherence of sequentially viewed words, irrespective of their syntactic congruency. This hemispheric specialization in semantic and syntactic processing could account for the observed differential patterns in semantic priming for words and syntactic priming for nonwords.

Such asymmetric processing capabilities between the hemispheres offer a plausible explanation for the divergent priming effects observed in our study. Furthermore, these strategic differences between the two cerebral hemispheres may result in a processing approach consistent with serial models. The hemispheric dissimilarity in semantic processing, especially the RH’s ability to generate significant semantic priming despite syntactic incongruence, contributes to the emergence of semantic priming effects in the face of syntactic incongruence. This supports a processing framework aligned with serial models rather than parallel models.

## Conclusions

Our findings revealed a hemispherically differentiated pattern of semantic priming. Specifically, the RH demonstrates robust semantic priming even in the presence of syntactic incongruence between the prime and target, indicating a strong activation of semantic attributes independent of syntactic relationships. Conversely, the LH, as the primary locus for syntactic processing, exhibits a form of processing dissonance when confronted with syntactically incongruent yet semantically congruent word pairs, manifesting in slower RTs compared to conditions where both semantic and syntactic elements are congruent.

These divergent patterns of semantic and syntactic processing across hemispheres may be attributed to inherent specializations in each hemisphere for these respective cognitive functions. Furthermore, our examination of nonwords suggested a distinct hemispheric pattern for syntactic processing, likely stemming from the absence of lexical entries for nonwords as opposed to words. The LH appears to be principally engaged in syntactic processing and may not necessitate substantial contributions from the RH, resulting in a lack of dynamic interhemispheric interactions and yielding significant intrahemispheric syntactic priming confined to the LH.

One limitation of the present study was that it assessed parafoveal responses exclusively within the context of a lateralized lexical decision task, which does not emulate natural reading conditions. Consequently, this constrained the generalizability of our findings. In future studies, it is imperative to examine the distinct primary processing proclivities of each cerebral hemisphere, specifically whether the LH predominantly engages in syntactic processing, or if the RH principally focuses on semantic operations during natural reading. One methodological suggestion to explore this inquiry involves the manipulation of semantic and syntactic relational elements within a word sequence. By utilizing eye-tracking methodology to evaluate real-time responses, we can attain a understanding of hemispheric asymmetry with respect to semantic and syntactic processing during natural reading. Such an approach would offer a more ecologically valid assessment compared to lateralized word presentation paradigms currently employed.

## Data Availability

The datasets generated and analyzed during the current study are available on https://www.kaggle.com/datasets/user138833/semantic-and-syntactic-processing.
